# Long-term self-treatment with methadone or buprenorphine as a response to barriers to opioid substitution treatment: the case of Sweden

**DOI:** 10.1186/s12954-015-0037-2

**Published:** 2015-02-18

**Authors:** Torkel Richert, Björn Johnson

**Affiliations:** Department of Social Work, Malmö University, Malmo, Sweden

**Keywords:** Illicit use, Heroin, Methadone, Buprenorphine, Self-treatment, Opioid substitution treatment, Barriers to treatment

## Abstract

**Background:**

It is well known that illicit use of methadone and buprenorphine is common among people with an opioid dependence. Less notice has been taken of the fact that these substances are also used for extended periods of self-treatment, as a way of handling barriers to OST. In this study, motives for self-treatment are investigated, as well as attitudes and perceived barriers to OST among drug users with an opioid dependence in Sweden.

**Method:**

The study is based on qualitative research interviews with 27 opioid users who have treated themselves with methadone or buprenorphine for a period of at least three months.

**Results:**

The duration of self-treatment among the interviewees varied from 5 months to 7 years. Self-treatment often began as a result of a wish to change their life situation or to cut back on heroin, in conjunction with perceived barriers to OST. These barriers consisted of (1) difficulties in gaining access to OST due to strict inclusion criteria, limited access to treatment or a bureaucratic and arduous assessment process, (2) difficulties remaining in treatment, and (3) ambivalence toward or reluctance to seek OST, primarily due to a fear of stigmatization or disciplinary action. Self-treatment was described as an attractive alternative to OST, as a stepping stone to OST, and as a way of handling waiting lists, or as a saving resource in case of involuntary discharge.

**Conclusion:**

Illicit use of methadone and buprenorphine involve risks but may also have important roles to play for users who are unwilling or not given the opportunity to enter OST. A restrictive and strict rehabilitation-oriented treatment model may force many to manage their own treatment. More generous inclusion criteria, a less complex admission process, fewer involuntary discharges, and less paternalistic treatment may lead to increasing numbers seeking OST. Control measures are necessary to prevent diversion and harmful drug use but must be designed in such a way that they impose as few restrictions as possible on the daily life of patients.

## Background

### Introduction and aim

Methadone- or buprenorphine-based opioid substitution treatment (OST) is a well-established treatment with positive effects on mortality, social and health problems, and criminality among people with an opioid dependence [1-3]. However, the medications used in the treatment also have a high abuse potential and are sought after on the illicit drugs market [4,5]. In several countries, diversion—the selling or sharing of medication—has been highlighted as a serious problem, with reference to (among other things) methadone-related deaths outside the treatment [6-10].

Illicit use of methadone and buprenorphine may bring health-related and legal issues but may also bring certain advantages. According to a study by Harris and Rhodes [11], illicit methadone use may serve as a ‘protection strategy’ enabling people with a drug dependence to control their drug use, improve their social relations, and protect themselves against hepatitis C. Illicit use of buprenorphine can also reduce health risks and improve quality of life compared to heroin use [5,12].

Illicit methadone and buprenorphine are mainly used by people with an established opioid dependence, often for various therapeutic purposes: to avoid withdrawal symptoms, for self-detoxification, to avoid heroin use, or as an analgesic [13-15].

Prevalence levels, user populations, and motives for using the substances vary from country to country. This may be explained by the differences in access to heroin and other opioids and by the differentiated pricing levels for various substances but also by the access to and the design of OST [5,16]. Some studies have pinpointed barriers to OST as an important explanation as to why users are self-medicating with methadone or buprenorphine. Such barriers may be associated with difficulties in gaining access to treatment [17,18] or with issues such as the treatment services not attracting or retaining opioid-dependent people to a sufficient degree [19-21].

Little is known about why opioid-dependent people refrain from seeking OST, how they perceive and handle existing barriers, as well as the significance of illicit use of methadone or buprenorphine in this context. The aim of this study is to investigate attitudes and perceived barriers to OST, the various motives for self-treatment, and the functions which self-treatment may serve for opioid-dependent people in Sweden.

The study is based on qualitative interviews with people with experience of self-treatment using methadone or buprenorphine for an extended period. We define ‘long-term self-treatment’ (henceforth ‘self-treatment’) as a daily or near-daily illicit use of one of the substances for a continuous period of at least 3 months.

### Opioid substitution treatment in Sweden

The Swedish OST model has been described as restrictive and rehabilitation oriented. The inclusion criteria are strict, treatment is often highly structured and controlled, and the dosage levels are relatively high. OST may only be administered by specialist psychiatric healthcare. Retention levels are typically high, and treatment has a clear focus on abstinence from drugs, rehabilitation, and lifestyle change [22-25].

Two circumstances in particular have had a great impact on the development of the Swedish OST model. Firstly, for a long time, there existed a vehement ideological resistance to OST in Sweden. This resistance resulted in a limited access to treatment, rigorous inclusion criteria, in addition to strict regulations and controls while in treatment [25-27]. Secondly, healthcare and social services in Sweden have joint mandatorship of the addiction treatment system. This has led to a clear separation of social and medical interventions, while simultaneously laying great stress on the importance of cooperation. For service providers in Swedish OST, the treatment’s legitimacy rests on factors such as therapeutic intervention; in doing more than just reducing harm. The general view is that OST should be aimed at social rehabilitation and ‘normalization,’ which means living a life ‘like most people do,’ free from drug abuse and integrated in mainstream society [25,28].

National regulations are issued by Socialstyrelsen (The Swedish National Board of Health and Welfare). The rules are strict and exclude populations which would have been offered OST in many other countries. The current regulations [29] have been in force since 2010 and stipulate that OST may only be given to people over the age of 20 with at least 1 year’s documented opiate (heroin, morphine, or opium) dependence. Users who primarily are dependent on other opioids, such as buprenorphine, fentanyl, or methadone, may not be offered OST. Nor may treatment be offered to users who are poly-dependent on other narcotic substances or alcohol, which pose a ‘substantial medical risk’. Medication is supposed to be administered under daily supervision for the first 6 months or for longer if deemed necessary. Regular urine tests are taken to check that no prohibited substances are used.

The regulations also outline a number of circumstances when treatment should be suspended: if the patient is absent from treatment for more than 1 week, repeatedly relapses into illicit drug use, uses alcohol in a way which constitutes a medical risk, manipulates his/her urine samples, or if he/she is convicted of drug offenses. If a patient is discharged, a 3-month suspension period is initiated, during which he/she is barred from seeking re-admission to OST.

The current regulations are a result of a gradual movement toward a less restrictive treatment policy over the last decade. Prior to 2005 at least 4 years of documented intravenous opiate addiction, in addition to three failed attempts—documented in medical records—at drug-free treatment were required in order to qualify for OST. Prior to 2010, collaboration between healthcare and social services was mandatory—anyone who applied for OST was expected to have a treatment plan in place with the social services. The suspension period in the event of involuntary discharged was two years in the 1990s but was reduced to 6 months in 2005 and further lowered to 3 months in 2010 [30].

Prior to 2005, access to OST was strictly limited, leading to long waiting lists, often several years [31]. After the new regulations [32] were issued, however, access to treatment increased rapidly. On a regional level, the changes toward a less restrictive model have continued since the introduction of the 2010 regulations. Involuntary discharges have become less frequent, particularly in Stockholm and Scania, where the majority of the Swedish patients are to be found (ongoing review by Socialstyrelsen).

Although the Swedish OST system has become more inclusive, substantial numbers of opioid-dependent users remain outside the treatment system against their will, mainly as a result of the rules banning specific opiates, the exclusion criteria, and the suspension period. In addition to this, some healthcare regions have long waiting lists and a protracted and arduous admission process^a^.

## Methods

### Participants and recruitment

The interviewees in this study were recruited as part of a comprehensive research project about diversion and illicit use of methadone and buprenorphine in Sweden. In this project, structured interviews were conducted with 411 OST patients in five towns and cities in the South of Sweden—the locations were selected in order to cover a variety of local drug scenes and varying access to OST—and with 98 opioid users at the Malmö needle exchange.

Posters and scheduling lists were put up at the various sites. We then spent from two to ten workdays at each OST program and several weeks at the needle exchange, carrying out scheduled interviews and recruiting additional participants among the visitors. A more in-depth description of the participants and recruitment process can be found in one of our previous articles [33].

One part of the interview dealt with experiences of methadone or buprenorphine use outside treatment and covered the procurement of the substances, manner of intake, as well as the extent and the motives for the usage. We soon discovered that it was common among the interviewees to have used one or other of the substances illicitly for an extended period—sometimes for years—as a means of self-treatment. This gave us the idea of conducting a specific study of the phenomenon.

In the quantitative study, 32% of the interviewees stated self-treatment as their primary motive for illicit use. Of them, around 30 people were offered the opportunity to participate in an in-depth qualitative interview. The objective was to create a broad and varied study population in terms of factors such as gender, age, history of drug use, and life situation. Furthermore, we wanted the population to include both users with a current self-treatment and patients enrolled in OST, who previously had managed treatment on their own. A total of 27 people agreed to participate in the study. All of them met the following inclusion criteria: (1) current or previous illicit drug use, primarily opioid-based, (2) experience of self-treatment with methadone or buprenorphine for a continuous period of at least 3 months, and (3) at least 20 years of age.

### Procedure

The interviews were carried out by Johnson and Richert. Before the interviews began, we informed all interviewees verbally and in writing about the project and its aims. We explained that the study was completely confidential and independent from the treatment programs, that participation would not affect their individual treatment, and that they had the option of ending the interview at any time.

The in-depth interviews were normally conducted following on directly from the structured interview, but in some cases we scheduled a new interview. Several interviews took place in a separate room at the OST clinics or in an interview room at the needle exchange. Some interviews were conducted in the home or workplace of the interviewee.

The interviews were topic based, but the interviewees were given ample opportunity to talk freely. The topics covered issues such as current life situation, experiences of drug use, previous treatment experiences, as well as experiences of using methadone and buprenorphine outside treatment. Particular attention was paid to the motives for self-treatment, attitudes to OST, and perceived barriers to OST.

All the participants in the study were offered a gift voucher worth SEK 200 (approximately EUR 22) or a book, regardless of whether they completed the interview or not. The interviews lasted between 30 and 90 min, and in a couple of cases supplementary interviews were conducted. A total of 11 interviews were recorded and transcribed verbatim. During the remaining 16 interviews, where the interviewees asked not to be recorded, we took notes which were typed up afterwards.

### Analysis

The analysis was performed as a manual, three-step qualitative textual analysis. First, we did a close reading of the material and performed a summary coding based on the overarching themes in the interview guide. Then, we made a second more detailed coding, where we identified similarities and differences in relation to the original themes. Finally, we went through the material one last time in order to identify suitable illustrative and representative quotes.

### Ethics

As already noted, the study forms part of a larger research project into diversion and non-medical use of methadone and buprenorphine in Sweden. The project was conducted in accordance with The Swedish Ethical Review Act (SFS 2004:460). The design and execution of the project was approved by the Regional Ethical Review Board at Lund University. All names used in this article are fictive, and any data that could reveal the identity of interviewees has been omitted.

## Results

### Sample characteristics and pathways to self-treatment

Twenty males and seven females, aged between 24 and 53 years, participated in the study. All of them were experienced drug users with at least 4 years of regular opioid use. Heroin had been the primary drug for 24 interviewees, while the other three had mainly used other opioids. All had also used several other types of substances.

At the time of the interview, 13 people had a current self-treatment. The remaining 14 were admitted to OST, meaning that their self-treatment dated back some time. A total of 20 people had some experience of OST. The duration of the self-treatment varied between 5 months and 7 years, but in the majority of cases it had lasted between 1 and 2 years. A total of 13 patients had primarily used buprenorphine, and 14 mainly methadone, although nearly all had experience of both substances.

There was considerable variation within the group in terms of life situation, as well as extent and consequences of drug use. Some described very severe drug problems and a life situation characterized by housing problems, support strategies based on crime, and marginalization. Others described how they, despite drug use leading to physical dependence, had been able to lead a relatively well-structured life with stable housing and work. In some cases, their drug use had been hidden from family, friends, and colleagues.

The interviewees described a number of pathways into self-treatment. All had first come across methadone or buprenorphine outside treatment, often as a means of alleviating heroin withdrawal symptoms, for self-detoxification purposes, or as a result of being unable to get hold of heroin. Often, first-occasion use was of short duration, a few days to a week.

More extended self-treatment was initiated at a later stage. For nearly everyone this had happened at a point when they were trying to quit or cut back on heroin use, in order to change their life situation. The impetus for change often was the culmination of a number of negative consequences of the drug use. In other cases, it had come in connection with a positive life event, such as starting a new relationship, becoming a parent, or an offer of employment. The transition to methadone or buprenorphine nearly always resulted in a reduction in heroin use and an improved life situation. In particular, the reduced outlays for drugs, as well as the long action of the substances, were seen as great advantages.

The interviewees described various attitudes to OST and different reasons as to why they managed treatment on their own. Three major explanations for self-treatment emerged: (1) difficulties in getting access to OST, (2) difficulties in remaining in OST, and (3) ambivalence about or reluctance to enter OST.

### Difficulties in gaining access to OST

#### To qualify and prove that you are a ‘genuine addict’

Having been denied OST was a common motive for starting self-treatment. Several interviewees described how their application for OST had been rejected since they did not fulfil the inclusion criterion of 1 year’s (alternatively 4 years’) documented opiate dependence. Some of the interviewees had hidden their drug use from friends, family, and colleagues and lacked documentation in the form of contacts with healthcare, dependence treatment, social services, or the police. Others were denied treatment since they were primarily dependent on synthetic opioids rather than opiates, which as previously noted is a requirement to get access to OST in Sweden. Other reasons for applications being rejected were excessive polydrug use (often in the form of sedatives), an unclear residence situation, or an impending prison sentence. Some explained that they did not even get to the stage where they could submit a formal application to the addiction treatment services, since they were dissuaded or prevented from applying by another authority^b^.

One example is Hamza, a young man aged about 25, who at the time of our interview recently had been admitted to OST for the first time. Hamza started using drugs and pills in his early teens and had been dependent on benzodiazepines and various analgesics, in particular oxycodone, for several years. He had also used heroin on and off. Hamza described how he previously had had a very difficult life situation characterized by mental ill health, uncertain residence situation, and an extensive polydrug use which resulted in several overdoses. In recent years, he had managed to improve his social situation by treating himself with methadone, which he bought from patients in OST. Asked why he had not applied for OST earlier, he replied:

I’ve been thinking about it for years. But I haven’t been admitted before. I didn’t have enough documented opioid abuse. They [the addiction treatment services] want it clearly to show that you’re addicted to heroin, which I’ve never been […] The first time I suggested substitution treatment to the social services, they just snorted derisively. They said that wasn’t for me, you’re not a junkie, you’re a polydrug user. They forced me into compulsory treatment […] There’s hardly anything about heroin in my records, but I’ve had several serious methadone ODs, which was probably the reason why I was finally admitted to treatment. They’ve started to relax the strict regulations a bit in recent years.

In Hamza’s case, the social services formed a first ‘border guard’ to OST. Since he applied for treatment before 2010, he had to go via the social services. The example also shows how the addiction treatment services initially denied him treatment because he lacked ‘proper’ documentation, only to admit him at a later stage, thus ignoring the criterion of admitting only people with a documented opioid dependence. Presumably, this was motivated by the fact that Hamza had overdosed on several occasions. The example indicates that, in the meeting between an applicant and official, there exists a certain amount of leeway when applying the regulations.

Another interviewee who encountered obstacles to OST is Peter, a man in his 40s, who has self-medicated with methadone for the last few years. Peter previously had a long-standing heroin dependence, although hidden from colleagues, friends, and family. He had had a relatively well-structured life with permanent residence and work and had never been in treatment. Already at an early stage of his heroin dependence, Peter had been motivated to enter OST, but when he was told by the social services that he had to present extensive documentation proving that he was a heroin addict, he let the idea go. Instead, he bought methadone illicitly.

I mean, I remember when I was fairly new to heroin, that I asked about substitution treatment and stuff. Yeah, are you an injecting drug user, that sort of thing? Well, I mainly smoke heroin, and I’ve been doing it for more than a year. Right, so you haven’t got any needle marks, then? No, no. Have you ever been arrested by the police or something like that? No, no. Okay, not been caught shoplifting or something? I respond ‘No, no.’ So, you can’t prove that you’re an addict? Then I’m sorry, we can’t offer you treatment. And that was about five years ago or so. So, I said, how am I going to get treatment then, you know? Do I have to commit a crime or something to get treatment?

Peter described how he had to prove that he was a ‘hard-core addict’ in order to get access to treatment, which in this case meant exhibiting needle marks or documentation proving a criminal record. Whether he fulfilled the criteria for opioid dependency was not even discussed. This example indicates that the attitudes of professionals and their norms as to who belongs to the ‘right category’ of drug user may influence an applicant’s chances of being admitted to OST.

#### Limited access to OST and an arduous admission process

Even people who fulfil all formal criteria for OST may encounter difficulties in getting access to treatment. Several interviewees, in particular those who had sought help some years back, pointed out that long waiting lists had meant that they were forced to wait a year or more for treatment. Some described the assessment process as so drawn-out, complex, and demanding that they had felt the need to initiate their own treatment.

Marc is a young man who, at the time of the interview, recently had been admitted to OST, after a second, drawn-out assessment. In total, he had waited more than 2 years. Most of this time, he had been using illicit methadone or buprenorphine for self-treatment purposes. The first time he sought help, he gave up after more than a year on the waiting list and instead started buying buprenorphine from acquaintances in OST.

I thought it all took too long. Everything took several months. I mean, it took three months just to get this appointment (the first information meeting), and then it took another three months before I saw the doctor, I hadn’t even given a urine sample, you know, so I was thinking like, how long is it gonna take? Shit, I’ll die before I… I was up there nagging them, I called and talked to them and so on, and they were like ‘yes, but that’s all we can do right now’ and stuff, and ‘it’ll take the time it takes’.

Eva had a similar tale to tell. She was 26 years old and had been in OST for 3 months when we interviewed her. Prior to this, she had used illicit methadone and buprenorphine for nearly 4 years, both as part of polydrug use and as a means of getting out of a longstanding heroin addiction. She found the road to OST difficult and exhausting.

I wanted treatment when I started using buprenorphine^c^ on my own, but I just didn’t get around to it. I was too deep in the shit, it was too chaotic […] After a while, I sought treatment anyway, but it was so messy,’ coz I was taking loads of benzo, heroin and speed, and I’ve got ADHD as well, it was insane. I missed appointments, and couldn’t get a grip. So, I started self-medicating with buprenorphine, tried to get off heroin, and to quit benzo and speed. About six months after I’d started with buprenorhine I managed to get enough structure and harmony to be able to seek treatment again. After that it took six months before I was admitted to treatment, and got buprenorphine legally. During those six months I took care of my own treatment.

Eva’s experiences are similar to those of many others. Several interviewees reported that it had taken more than a year from the point where they applied for OST until they actually began OST. In a number of cases a treatment plan from the social services was required before the person was referred for assessment by the addiction treatment services, in particular for those who applied for treatment before 2010.

At the clinic, new patients are invited for an information meeting where the treatment staff establishes whether the criteria for OST are fulfilled or not. From then on, ongoing contact is still required just to remain on the waiting list. Overlooking a letter or missing an appointment in some cases means losing the place on the waiting list. Before treatment begins, the clinic often demands detoxification, not infrequently, causing further delays. Many interviewees characterized the assessment process as one long obstacle course with unreasonable demands.

The examples above all illustrate various difficulties in gaining admission to OST. The interviewees describe something akin to a Catch-22 situation. In order to gain admission to OST, the applicant on the one hand has to show zeal and have a sufficiently ordered life situation to cope with a long and arduous assessment process. On the other hand, the individual is required to prove that he or she is a ‘hard-core addict,’ by providing documented negative consequences in the form of overdoses, previous failed treatments, drug crimes, and needle marks. Anyone with a hidden or insufficiently documented drug use runs a great risk of ending up outside the system. This applies also for people with a very difficult and chaotic life situation, extensive polydrug use, or mental health issues.

The examples in this section also illustrate the important roles that illicit methadone and buprenorphine may play. People who do not ‘qualify’ or who give in along the way can buy the substances on the street and manage their own treatment. For some, illicit medication serves as a bridge into treatment by giving them the opportunity to stay motivated or give them sufficient harmony and structure in life to be able to cope with the admission process.

### Difficulties in remaining in OST

Admission to OST is no guarantee for continued treatment. Patients not adhering to the rules of the programs run the risk of being discharged. As mentioned in the section about OST in Sweden, there are several official ‘just causes’ for an involuntary discharge [29]. According to the national regulations, a discharge carry an automatic 3-month suspension, during which the patient is barred from re-applying for OST. In practice, however, the waiting time is often considerably longer since waiting lists for the programs are common.

Involuntary discharge was a reason cited by several interviewees as to why they had started self-medicating, either while they were waiting for re-admission to OST or because they no longer wanted to remain in treatment. The most common discharge reason was continued use of illicit drugs, which is well in line with the results of previous Swedish research [34]. Missed clinic appointments, disorderly or threatening behavior, and drug crimes were also cited as reasons.

One interviewee with vast experience of self-treatment due to discharges is Sophie, a 53-year-old woman who at the time of the interview had begun her seventh OST episode. Sophie had been discharged six times, on each occasion due to relapses into drug abuse, mainly benzodiazepines. Several of the discharges had landed Sophie in a difficult situation; the most recent discharge, for instance, had resulted in her losing her housing provided by the social services.

The last three times Sophie was discharged, she bought illicit methadone in order to continue treatment on her own. It often took several months, in some cases more than a year, before she was re-admitted to treatment. Sophie characterized the discharges as if ‘they’d cut me down at the knees’ and maintained that the illicit methadone ‘had been her salvation’ during these periods.

Another example is Josef, a 29-year-old man whom we encountered at the needle exchange. A few weeks earlier, Josef had been discharged from treatment for the fourth time. He was now using methadone or buprenorphine, depending on what he was able to get hold of. He explained that he had difficulties in keeping his place in treatment, since he was seen as ‘disorderly’ and ‘troublesome’ by staff. ‘I’ve got ADHD, have difficulties controlling impulses, turning up on time, keeping appointments and so on, treatment doesn’t work for me,’ he told us. Josef was critical of the regulations and of the fact that he had been involuntarily discharged several times. In treatment, he felt powerless in relation to the staff, and in his view there ought to be more flexibility and greater consideration of his situation. ‘I feel you’ve got to have certain rights even if you’re an addict in treatment,’ he explained.

Another interviewee who pointed to strict regulations and the demand to stay off drugs as the impetus for turning to self-treatment is Anders, a 42-year-old man who recently had been released from prison, after serving a lengthy sentence for drug-related crimes. Anders had used heroin and cannabis regularly for more than 20 years and periodically also benzodiazepines, amphetamine, and cocaine. He used methadone and buprenorphine for self-medication purposes. At the time of the interview, he had just been admitted to OST for the first time, and he was about to start his methadone titration. He had previously avoided seeking treatment because he was convinced that he would be discharged quickly due to his cannabis use.

I know I’ll get problems in treatment because I smoke hashish more or less every day. I won’t give up this habit, I don’t want to. I’ve been smoking since I was 12, and I need it to feel okay. I don’t smoke much, but often a little every day. I’ll try telling them [treatment staff] how it is, that I smoke some hashish, and that I’ll test positive on the urine samples. We’ll see if they go along with it. To my mind they need to put this in it’s proper perspective. I mean, the important thing is for me to quit the junk, and serious crime and stuff. Then, for sure, it’s important not to do benzo or too much alcohol when going on methadone, I know that, that it’s dangerous to combine the substances. But to smoke some weed isn’t dangerous and shouldn’t prevent you from getting treatment. It’s not stopping me from studying or working either, I was able to do that before. If they can’t accept that I smoke, I’ll have to go back to self-medicating.

Anders’ account reflects two different attitudes to OST, attitudes that are reminiscent of what Ekendahl [25] defines as a normalization and a harm reduction discourse, respectively. On the one hand, Anders stressed the importance of giving up heroin and criminality, which is in line with the normalization discourse. He described smoking cannabis as legitimate, since he saw that as self-medication, and it had not prevented him from studying or working. On the other hand, he highlighted the importance of reducing harm from and negative consequences of drug use, rather than achieving complete abstinence, which is more in line with the harm reduction discourse.

The examples in this section show how a restrictive treatment model, combined with regulations urging discharges when rules are violated, both can lead to repeated discharges and deter some people from seeking treatment. This primarily seems to affect people who are in a difficult situation already, such as concomitant mental ill health or highly problematic drug use.

### Ambivalence or reluctance about entering OST

A number of interviewees admitted feeling ambivalent about OST or reluctant to seek this treatment. One reason for this could be that hitherto hidden drug use then would be assessed and revealed. Some perceived it as shameful having to seek help and as stigmatizing being labeled an addict. Some also disagreed with the controls and loss of autonomy which OST involves.

#### Fear of being labeled or exposed as an addict

Several interviewees pointed out that their drug use to a significant degree had been hidden from their nearest and dearest, and that they did not see themselves as ‘real junkies.’ Consequently, seeking treatment would be a delicate, mortifying matter.

An example of this is Micke, a 31-year-old man who had been in OST for about a month; but prior to that, he had put off seeking help—treating himself in the meantime—for more than a year. The main reason was that he did not want to openly admit his addiction and risk losing custody of his daughter.

The reason why I self-medicated the first time, with buprenorphine, was that I didn’t want to seek treatment. My addiction wasn’t known to many people at the time, neither by my colleagues nor by my family. Of course, I knew I needed treatment, and that it would make life easier for me, but I didn’t want to reveal my heroin addiction to the social services, because I would then risk losing custody of my daughter. That was a risk I wasn’t prepared to take.

Another example is Johan, a 27-year-old man, who discussed the perils of revealing his dependence on drugs but also his unwillingness to get in touch with the authorities and running the risk of being labeled an addict. At the time of the interview, he was using illicit methadone. He described how he had been self-medicating for more than a year, and that it had helped him quit heroin, improve his health, and that he had managed to get a job. He saw great advantages with self-treatment, since it enabled him to avoid contact with authorities, and he did not have to tell his family or his employer that he was a former heroin user. Most of all, he was worried that revealing his background would lead to him losing his driver’s license or his job.

So I like tried hiding it,’coz it was a bit humiliating, you know, to tell mum and the rest about the heroin […] I’ve never really liked involving the authorities and stuff, and get labeled. Most importantly because of my job, I’ve got important endorsements on my driver’s license and stuff.

Karin, a 32-year-old woman, also described it as negative having to be assessed and categorized as an addict. For her, it was less the fear of any immediate consequences and more about the feeling of shame and the threat this posed to her own self-image. Karin had been using analgesics, mainly morphine, for many years. She characterized her drug use as self-medication and did not see herself as a ‘real addict or junkie’. Karin had a well-paid job, owned her own home, and told us that she had not experienced any significant social problems because of her drug use. At the time of the interview, she was in OST but explained that she had put off seeking help for several years, because she felt ashamed about having to apply for treatment via the social services.

#### Freedom from control measures and authority

Virtually, all the interviewees voiced the opinion that OST subjects patients to control measures and authority, and some even characterized the treatment as degrading. In order to receive medication, you have to be in a certain place at a certain time, and nobody has an indisputable right to have time off. According to the regulations, the medication is supposed to be taken under supervision at the clinic during the first 6 months. Patients are not allowed to use other narcotic substances or to be under the influence of alcohol when collecting medication. Furthermore, regular urine samples are taken under supervision. Anyone who fails to adhere to the regulations risks being sanctioned, which in the worst case scenario means involuntary discharge. From this perspective, managing your own treatment can mean greater autonomy and a sense of independence and self-determination.

Self-treatment, however, is far from unproblematic. The high cost of illicit medication and the limited access to the substances were issues mentioned by several interviewees. A number of the interviewees explained that they had to pay several thousand Swedish kronor (hundreds of euros) every month for their medication^d^. It was relatively common for them to go without medication for one or more days, due to difficulties in finding a dealer, or because they were hard up. Difficulties in keeping drug use ‘under control’ was another issue that emerged in our interviews. Some interviewees told us how they had experienced such difficulties in managing their self-treatment that they, albeit very reluctantly, felt compelled to seek OST.

Tim, a man in his 40s, told us how he had treated himself with buprenorphine for many years. He explained how this previously had worked well, but that he recently had begun to lose control. Difficulties in getting medication and an accelerating polydrug use—heroin and benzodiazepines among other substances—led to a worsening social situation and several overdoses. Tim had never been in OST before, but at the time of the interview he had just applied for admission to a program, since he felt he needed help to control his drug use and straighten out his life. His justifications for self-treatment and his thoughts about what had prompted him to seek OST went as follows.

It’s about evading the control measures in there. You know, having to give supervised urine samples and that. Then they boss you around, telling you what you can and can’t do. If you’re feeling rough one day and you want to take a few benzos, you’re not allowed to. And then you have to go there every day for six months to collect the medication. Then, if you have been a good boy and kept to the straight and narrow, you’re allowed to take the medication home. But that’s just it, it’s these control measures that I want to avoid. I’ve been thinking about seeking treatment for ages, and I’ve come to the conclusion that maybe now’s the time. I mean, I’m always running around chasing [the substances]. You need it [buprenorphine] every day, so why not … and these control measures that we talked about earlier, but if I’m a good boy and I *want* to do it, then the controls aren’t that bad at the end of the day, not if it’s voluntary on my part too, if I decide that now I’m gonna take buprenorphine and stay on it, and try to sort out my life, the social side of things, working or studying.

Tim’s analysis indicates that treatment can be perceived as controlling, intimidating, and disciplinary. Tim really did not want to yield to the control measures but still appeared to have realized that he needed help. Moreover, he was tired of constantly chasing illicit medication. His analysis reflects a form of inner negotiation, where he is trying to convince himself that the control measures may not be that daunting after all.

One way of alleviating the negative experience of powerlessness and subjection is to redefine the demands on good conduct from a passive to an active position. To be good, well mannered, and well behaved in order to live up to the demands and expectations of the staff is different from behaving properly on your own accord in order to sort out one’s life and get a job. By redefining the situation, Tim redresses the power balance, at least intellectually. The perceived autonomy increases, and the regulations appear less terrifying.

Other interviewees considered the loss of control that treatment entailed too great a threat to their integrity. Max is a 45-year-old man who had previously been in treatment but had chosen to discontinue it due to negative experiences of control measures and disciplinary actions. In particular, he was critical of the travel restrictions (imposed by the treatment system), since he sometimes wanted to go abroad to see friends and family and for work purposes. At the time of the interview, it had gone 7 years since Max terminated his OST. From then on, he had managed his own treatment, using illicit methadone.

Max found great similarities between being a heroin addict and being an OST patient. To some extent, the patient is forced to associate with the same people (in the waiting room at the clinic), and the patient is dependent on a substance (medication instead of heroin) and on treatment staff instead of dealers.

So in a way, it’s the same thing as going on heroin. You’re always forced to turn to a dealer or a pusher, and in this case [the treatment staff of the program], it’s a freaking nuisance of a pusher, who tells you, you can’t smoke hashish, you can’t drop pills, you’ve got to be on time, and you know … You see what I mean?

Max preferred methadone to heroin, but he also preferred the power of the ‘methadone pusher’ over the power of the treatment system. According to him, managing one’s own treatment is the best of three bad options. It emancipates the user from the compulsions of life as a heroin addict—the constant money chasing, interspersed with brief moments of satisfaction—while also throwing off the shackles of the clinic, and its strict rules and control measures.

I mean, from having been an addict, with all that stuff completely controlling your life, that’s what you want to avoid, right … so, the whole idea of being ostensibly drug-free through medication, all that goes up in smoke with all those rules, you know. That’s the whole point, when you stop using drugs, you want that freedom, you see. And they put a stopper to it right away. […] The thing is, I can’t cope with it, maybe it’s just me, but I can’t do it. The way I see it, it’s like you’re giving up if you agree to those conditions.

Seeking treatment is often seen as an important step on the road to rehabilitation, getting a grip on life, to break free from the dependence, and change one’s life situation. The picture Max is painting is quite the opposite. To enter OST is for him a sign of surrender in the struggle for a life free from dependency and control measures.

## Discussion

### Illegal use of OST medication—benefits and risks

Illicit use of methadone and buprenorphine has been characterized as something negative and risky, both in research and in the drug policy debate [5,8]. In Sweden, an increase in methadone-related deaths in recent years have been associated with diversion of medication from OST programs. The expansion of OST and the new less restrictive regulations have been identified as a purported cause [35]. Critics of OST have also maintained that illicit buprenorphine has become an increasingly common street drug among young drug users [36].

Although illicit use of OST medication may carry health, social, and legal implications, it is important to present the complexity of the issue, both of the illicit use itself, and of the forces driving the black market for the substances.

A current study indicates that diversion from Swedish OST programs is a relatively common phenomenon [33]. Another recent study, however, demonstrates that illicit buprenorphine is used only very rarely by adolescents and young adults whose drug use is not already problematic [15]. The study indicates that the illicit use of these substances primarily occurs among opioid-dependent users outside OST, often for self-medication purposes, which is in line with several other studies [13,37-40].

Another study, which focused on the attitudes to and views on diversion and illicit use among OST staff, also gave a multifaceted picture of these phenomena [41]. Although the staff saw diversion and illicit use as something negative and risky—the risk of fatalities through overdosing if the substances reaches people with no opioid tolerance, and the risk of diversion damaging the legitimacy of treatment were factors stressed in particular—illicit use nonetheless was considered a better option than using heroin. Staff members claimed that nearly all patients who begun OST had some experience of using the substances illicitly. Furthermore, patients who had used them as a means of avoiding heroin often were in a ‘better state’ than those patients who had mainly used heroin [41].

The above mentioned studies all point to insufficient access to OST as a partial explanation of the illicit market for methadone and buprenorphine. This is something which also comes across in the accounts of our interviewees. This study shows that illicit methadone and buprenorphine are often seen as better alternatives to heroin. These substances can give people with a heroin dependence an opportunity to cut back on their heroin consumption and improve their life situation. They can also be used for long-term self-treatment—as an alternative to OST, as a stepping stone to OST, and as a way of handling waiting lists, or as a saving resource in case of involuntary discharge.

The positive aspects of illicit use singled out here, should not in any way be interpreted as diminishing the issues and risks associated with such use; the substances are used for other purposes than self-treatment, and in riskier ways than those described by our select population of very experienced drug users. For anyone lacking tolerance for opiates, or with limited knowledge of methadone and buprenorphine, these substances can be lethal [42,43].

The results of our study reveal advantages with illicit use of OST medication compared to heroin use, but also that self-treatment may bring problems, not least in the form of limited access to medication, and difficulties in regulating the medication dosage^e^. One conclusion to be drawn from this is that it is crucial to lower barriers to OST, and to include more users with an opioid dependency in treatment. This would reduce their vulnerability, and potentially also reduce the demand for OST medication on the illicit market.

### Restrictive policies and negative attitudes to OST act as barriers to treatment

OST can be seen as a discursive field where science and values shape regulations, organization and practice jointly [25,44]. As mentioned previously, the Swedish OST model should be understood in light of the vehement ideological resistance which previously existed in the country. For a long time, OST was seen as a last resort, only to be offered to the hardest hit population of heroin users [25,26]. The previous inclusion criteria with mandatory documentation proving 4 years of intravenous opioid abuse and several failed attempts at drug-free treatment were direct results of this.

According to Swedish drug policy, solutions to drug abuse are supposed to be firm, thorough, and abstinence oriented [45,46]. For this reason, OST has been seen as a measure which should aim at rehabilitation and normalization, rather than harm reduction [25]. Abstinence and normalization have always been vital goals of the treatment and have legitimized a practice with frequent, supervised urine samples [30]. Although treatment has become less restrictive and more tolerant of relapses in to drug use in recent years, the current treatment policy still presents a number of barriers to OST.

Judging by the accounts of our interviewees, the current inclusion criteria, long waiting times, and an often arduous and bureaucratic admission process, in conjunction with the preconception of professionals of who belongs to the proper drug user category constitute such barriers. Negative attitudes toward OST, fear of stigmatization, and disciplinary actions raised further barriers to seeking OST.

There are mainly three groups of opioid users who appear to be affected by these barriers. People dependent on synthetic opioids, rather than opiates, do not fulfill the inclusion criteria according to the current regulations. People with a hidden and/or undocumented opioid dependence encounter difficulties in qualifying for treatment and often experience the assessment process (and previously, the mandatory contacts with the social services) as intimidating or stigmatizing. Anyone with a chaotic or precarious life situation—for instance users with severe mental ill health and/or an extensive polydrug abuse—often have difficulties in coping with an arduous admission process, as well as keep the mandatory daily appointments at the clinic and staying drug free.

There is previous research which indicate that restrictive treatment regimes may severely limit access to treatment [16-18], and that fear of stigmatization and restrictions imposed on daily life act as barriers to OST for many drug users [19,47]. Patients’ concerns about the ability to adhere to the treatment rules and ability to remain in treatment have also been put forward as a reason for not entering treatment [20].

The negative attitudes to OST displayed by our interviewees are based on their own experiences, as well as the views they have formed through the interaction with other drug users. Previous research has also shown how drug user’s attitudes to OST are influenced by norms and values within ‘the addict subculture.’ This research indicates that the drug life on the street is often seen as more active, independent, and exciting than the neatly ordered and controlled life as a patient in OST [48-50].

OST has been characterized as a treatment which ‘takes your heart out,’ and methadone patients are sometimes considered ‘losers’ by other drug users, since they are no longer seen as being able to cope with the more challenging and demanding life as a heroin user [49]. In another study, methadone patients characterized their situation in terms of stigmatization and a marginalized identity—as patients, they were not considered ‘genuine addicts’ anymore nor were they seen as ‘normal’ or drug free [51].

Our interviewees painted a partly different picture. Several interviewees explained that both ‘heroin life’ and life in OST involve restrictions and voiced the opinion that self-treatment offered greater autonomy and freedom—freedom from controls and restrictions of the OST clinic but also freedom from the constant money chasing of heroin life, and the precariousness which often characterizes this type of lifestyle.

Harris and Rhodes [11] argue that a less restrictive OST may have a liberating potential, if the patients are given greater control over their own lives, and opportunity to develop their own strategies to improve their health and social situation. Rigid constraints and supervised consumption can cause as well as reduce harm, they point out.

Some studies indicate that take-home doses achieve better outcomes than inflexible dosing and/or supervised consumption as regards retention rates, employment, and improved quality of life [52,53]. These studies, furthermore, suggest that take-home doses are not necessarily leading to an increase in crime, illicit drug use, or diversion.

Other studies, however, have pointed to control measures and supervision as having several important roles to play. Control measures have been suggested as a means of reducing illicit drug use among patients and have been linked with a decreased mortality associated with methadone diversion [9]. Control measures and rules may also serve as a support for patients in their rehabilitation process [54].

An important challenge for OST is to strike the right balance between control measures and freedom and between high ambitions and reasonable demands and expectations. As objectives, rehabilitation and abstinence can be positive, if they are realistic, designed in line with the patient’s own wishes and abilities, and are not coupled with sanctions. High ambitions may be beneficial for patients with good preconditions to live up to the demands or are offered the necessary help to do so. However, high demands and strict rules and inclusion criteria may also mean that some patient populations are excluded from OST or choose to manage treatment on their own.

Supervision and control measures are inevitable components of OST, and they should primarily be prompted by the objective of minimizing medical risks for the patients and the risk of the substances being diverted to the black market. It is important to find solutions that are both safe and acceptable for the patients—to ensure that programs expand access to and increase retention in treatment, without endangering them or compromising quality [20].

### Involuntary discharge: a questionable practice

Involuntary discharge from OST was a common motive for self-treatment among our interviewees. Some of the patients who had been involuntarily discharged described the access to illicit medication as lifesaving. In two previous Swedish studies [34,55], the practice of involuntary discharges has been characterized as dubious and inhumane since it nearly always increases health risks and bring about other negative consequences for anyone who is discharged. Among other things, discharges have been linked to a significantly increased mortality [56,57], and the substances used in the treatment may cause strong withdrawal symptoms if treatment is discontinued. Criticism has been leveled at both the national regulations, which cite a number of circumstances when discharges should take place and the not infrequently arbitrary nature of discharge decisions by staff [55].

Discharges pose a dilemma for OST. While discharges in the majority of cases worsen the life situation for the patient in question, there are also valid arguments for having the option to discharge or at least reassign a patient to another program. Patients who neglect or mismanage their treatment not only put themselves at risk, their conduct can also have a negative impact on treatment practice and the legitimacy of treatment [41]. Patients who use benzodiazepines or alcohol while being treated with methadone risk suffering an overdose. Patients who systematically sell their medication to people outside treatment put other people’s lives at risk and may also besmirch the reputation of the treatment. Patients who turn up at the clinic strongly intoxicated or behave threateningly toward staff or fellow patients create an unsafe and destructive treatment environment.

The attitude to the overarching treatment aims is of relevance when evaluating the practice of involuntary discharges. There are, as already noted, two principal approaches. From a normalization perspective, with its strong focus on rehabilitation and abstinence, discharges can be legitimized on other grounds than the purely medical; for instance, if the patient is not fulfilling his/her commitments or is breaking the program rules [55]. From a harm-reduction perspective, where the focus is on reducing drug use and its negative consequences for the individual, discharges are harder to justify since they nearly always put the patient at greater risk than if he/she were to remain in treatment.

The Swedish treatment model has traditionally been based primarily on the normalization perspective, although in recent years harm-reduction has gradually grown in importance. Involuntarily discharges due to transgressions have been common, also in cases where the misconduct has not brought any clear medical risk for the patient. Cannabis use, absence from treatment for some time, as well as disorderly behavior are examples cited by our interviewees^f^.

The opportunities for patients to express resistance are limited. Hirchman’s [58] terms ‘exit’ and ‘voice’ throw light on two possible resistance strategies for dissatisfied service users. ‘Exit’ refers to the option of leaving one service organization for another, while ‘voice’ means to speak up and protest. In Sweden, it is typically not possible to switch programs^g^, and speaking up and voicing criticism is, according to many patients, fraught with perils [34]. This means that patients find themselves at a considerable disadvantage in relation to staff [50,55]. The dependency on medication and the suspension rule exacerbate this disadvantage. For anyone critical of the control measures or unable to live up to the demands associated with regular treatment, self-treatment with illicit medication may be the only available option.

Involuntary discharges are both ethically and medically questionable. Discharges may be unavoidable under certain circumstances, but in our opinion this practice ought to be applied extremely sparingly and only after other reasonable measures have failed. A discharge should not prevent anyone from quickly receiving a place in another unit or operation. The suspension period appears to be a Swedish phenomenon; there is no scientific support for this practice, and it goes against research findings which indicate the importance of high retention and low thresholds to treatment [20].

## Conclusion

The way OST is organized in Sweden means that many people with an opioid dependence end up outside the treatment system, despite the fact that they want to reduce their drug use. Some of them decide to manage treatment on their own, using illicit methadone or buprenorphine. For the majority of our interviewees, self-treatment opened up an opportunity to cut down on their heroin use and improve their life situation but was also fraught with difficulties. An important objective, therefore, ought to be to lower the barriers to OST. Shorter waiting lists, more generous inclusion criteria, and simplified admission processes could contribute to this, as would fewer involuntary discharges, an abolished suspension period, and a less paternalistic treatment practice.

The high ambitions of the Swedish OST model, aimed at normalization and abstinence, may be a force for good if they are worked out in partnership with the patient and if they are not leading to unreasonable demands and an increased risk of involuntary discharges. Control measures and regulations are necessary for the treatment, but they should first and foremost be aimed at reducing diversion and harmful drug use. Control measures should be designed in such a way that they impose as few restrictions as possible on the daily life of patients and without appearing intimidating or degrading.

## Study limitations

The interviewees involved in this study constitute a select group of opioid users. All of them have encountered various barriers to OST, and many have negative experiences of OST. This means that they are decidedly more critical toward OST than most other patients we have interviewed in our project on diversion and illicit use of methadone and buprenorphine. It should be pointed out that many patients have positive experiences of OST and do not find treatment degrading or stigmatizing.

The participants in this study are, furthermore, a particular population in terms of motives and experiences of illicit use of methadone and buprenorphine. They all have had the ambition to reduce their heroin use and change their life situation and have primarily used the substances for these purposes. There are also people who use the substances in other riskier ways and for other purposes than self-treatment.

The accounts of our interviewees are based on current experiences as well as experiences that may be several years old. Thus, some of these experiences reflect past issues with the Swedish OST system, problems which may be less prevalent today.

## Endnotes

^a^Prior to admitting a patient, a social and medical assessment is required, which involves several meetings. Often, the person is required to go through detoxification before the medication titration process commences.

^b^For many years in Sweden, prior contact with the social services was a mandatory requirement for anyone applying for OST. During the period 2005–2009, this was a requirement in the regulations. The formal requirement was rescinded when the current regulations (SOSFS 2009:27) came into force, but many OST programs still require the contact to be initiated through a referral from the social services.

^c^Most of our interviewees use the medical product name, Subutex or Suboxone, but in the quotes we have altered this to the substance name throughout.

^d^The average price of illicit methadone and buprenorphine varied depending on location (the study was conducted in several towns and cities). For 90 mg of methadone, the price varied between SEK 230 and SEK 360 (approximately EUR 25–40), for 8 mg of buprenorphine between SEK 110 and SEK 280 (approximately EUR 12–31), and for 8 mg buprenorphine-naloxone between SEK 90 and SEK 250 (approximately EUR 10–27). The dosage used in self-medication tended to be significantly lower than the dosage used in official treatment programs, where the average 24-h doses are 100 mg methadone, 18 mg buprenorphine, and 19 mg buprenorphine-naloxone, respectively.

^e^The way the interviewees managed their self-treatment, how they got access to their medication, and the various problems they experienced when self-treating will be discussed in greater detail in a future article.

^f^It should be noted, however, that Swedish OST programs in general demonstrate relatively high retention rates [59]. In all likelihood, this is associated with the fact that strict inclusion criteria have created a select patient population, and that Swedish programs have comparatively high dosage levels. Another factor may be that waiting lists and suspension periods act as a deterrent for some patients, making them more scrupulous about keeping their places in treatment.

^g^However, a pilot project with free choice for OST is being launched in one region.
